# The TUSC2 Tumour Suppressor Inhibits the Malignant Phenotype of Human Thyroid Cancer Cells via SMAC/DIABLO Protein

**DOI:** 10.3390/ijms21030702

**Published:** 2020-01-21

**Authors:** Raffaela Mariarosaria Mariniello, Francesca Maria Orlandella, Anna Elisa De Stefano, Paola Lucia Chiara Iervolino, Giovanni Smaldone, Neila Luciano, Nara Cervone, Francesco Munciguerra, Silvia Esposito, Peppino Mirabelli, Giuliana Salvatore

**Affiliations:** 1 Dipartimento di Scienze Motorie e del Benessere, Universita’ “Parthenope”, Via Medina 40, 80133 Napoli, Italy; 2 CEINGE—Biotecnologie Avanzate S.c.a.r.l., Via Gaetano Salvatore 486, 80145 Napoli, Italy; 3 IRCCS SDN, Via Emanuele Gianturco 113, 80143 Napoli, Italy; 4 Dipartimento di Scienze Biomediche Avanzate, Universita’ “Federico II”, Via Pansini 5, 80131 Napoli, Italy

**Keywords:** thyroid cancer, TUSC2, migration, invasion, apoptosis, SMAC/DIABLO

## Abstract

Thyroid carcinoma is the most common endocrine cancer and includes different forms. Among these, anaplastic thyroid carcinoma (ATC) is the rarest but the most lethal subtype, compared to papillary thyroid carcinoma (PTC) which shows an overall good prognosis. We have previously showed that Tumor Suppressor Candidate 2 (TUSC2), a known tumour suppressor gene, is downregulated in human PTC and ATC compared to normal thyroid samples. The aim of this study was to gain insight into the molecular mechanisms induced by TUSC2 in thyroid cancer cells. Here, we stably transfected TUSC2 in papillary (TPC-1) and in anaplastic (8505C) thyroid cancer cell lines and studied its effects on several biological processes, demonstrating that TUSC2 overexpression decreased thyroid cancer cell proliferation, migration and invasion. Through the proteome profiler apoptosis array, we observed that TUSC2 increased sensitivity to apoptosis by increasing the SMAC/DIABLO and CYTOCHROME C proteins. On the other hand, transient silencing of TUSC2, by siRNA, in an immortalized thyroid follicular epithelial cell line (Nthy-ori 3-1) showed the opposite effect. Finally modulation of SMAC/DIABLO partially rescued the biological effects of TUSC2. Thus, our data highlight a tumour suppressor role of TUSC2 in thyroid carcinogenesis, suggesting that it could be a promising target and biomarker for thyroid carcinoma.

## 1. Introduction

Tumour suppressor candidate 2 (TUSC2), also known as FUS1, is located on the short arm of human chromosome 3 and behaves as a tumour suppressor in several human cancers [1]. The TUSC2 gene encodes a multifunctional protein that plays an important role in regulating a wide range of cellular processes, such as cell cycle arrest and apoptosis, in modulating the function of several kinases, such as EGFR, PDGFR, AKT and c-ABL, and in affecting gene expression [2,3].

Loss or reduction of TUSC2 has been reported in non-small cell lung carcinomas (NSCLC), in small cell lung carcinomas [4], in mesothelioma [5], in oesophageal carcinoma [6], in glioblastoma [7] and in sarcomas [8]. These observations corroborate the significant impact of this tumour suppressor gene in human carcinogenesis.

Emerging evidence suggests that restoration of TUSC2 could represent an attractive strategy to inhibit tumour growth and progression in cancer therapy. Deng and colleagues reported that forced expression of TUSC2 enhanced the antitumour activity of cisplatin in human lung cancer cells [9]; TUSC2 restoration sensitized lung cancer cells to treatment with EGFR inhibitors [10]. TUSC2 can inhibit mTOR activation [10] and potentiate tumour sensitivity to AKT inhibitors in NSCLC [11]. Importantly, the intratumoural administration of TUSC2 through a liposomal vector in lung cancer xenografts reduced tumour growth [12]. Finally, TUSC2 nanoparticles were administered intravenously, in clinical trials, in lung cancer patients [13] and showed safety and antitumour activity.

The vast majority of thyroid carcinoma (approximately 95% of cases) are derived from thyroid follicular epithelial cells, whereas the remaining 3–5% of cases originate from parafollicular C cells. Medullary thyroid cancer (MTC), originating from parafollicular C cells, is a neuroendocrine tumour that secretes high levels of calcitonin and is generally sporadic, even though 20–30% of cases are familial [14]. Thyroid carcinomas derived from follicular cells include papillary, follicular, hurtle cell, poorly differentiated and undifferentiated or anaplastic thyroid carcinoma. Undifferentiated or anaplastic thyroid cancer (ATC) is one of the most aggressive human tumours refractory to standard treatments with a very poor prognosis. ATC patients usually present a rapidly enlarging neck mass, a high rate of distant metastases and approximately 95% mortality at six months [15,16]. Conversely, well-differentiated papillary thyroid carcinoma (PTC), the most common type of thyroid cancer [17], is characterized by a good outcome as it is highly curable by surgery and radioiodine therapy; nevertheless some PTC patients have an aggressive disease and can develop distant metastasis [18]. Thus, novel treatment approaches are needed for these incurable thyroid carcinomas.

We have previously reported, by quantitative real-time PCR (q-RT-PCR) and immunohistochemistry analysis, that TUSC2 is downregulated in almost all ATC samples and in the vast majority of PTC samples, suggesting an important role of this tumour suppressor in thyroid cancer progression [19]. Nevertheless, the exact biological function of TUSC2 in thyroid cancer cell lines remains unexplored. 

Here, through functional experiments in PTC (TPC-1) and in ATC (8505C) cell lines, we showed that forced TUSC2 expression is able to inhibit cell proliferation, migration and invasion. On the other hand, transient silencing of TUSC2, by siRNA, in an immortalized normal thyroid follicular epithelial cell line (Nthy-ori 3-1) showed the opposite effect. In this context, we demonstrated that TUSC2 enhanced sensitivity to apoptotic treatment with doxorubicin (DOXO) or staurosporine (STS) and, through proteome profiler human apoptosis array and functional experiments, we unveiled that this effect was mediated in part by SMAC/DIABLO protein.

Taken together, these novel findings demonstrate that TUSC2 is negatively associated with thyroid cancer aggressiveness and thus this tumour suppressor could represent a novel potential target and biomarker for thyroid cancer therapy.

## 2. Results

### 2.1. TUSC2 Forced Expression Decreased Cell Proliferation in Thyroid Cancer Cells

**Figure 1:**
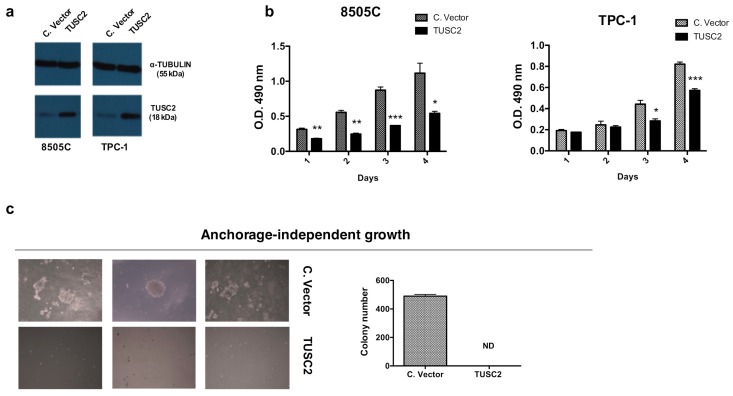
Tumor Suppressor Candidate 2 (TUSC2) forced expression reduced thyroid cancer cell proliferation. (**a**) Western blot analysis of TUSC2 and TUBULIN protein levels in a selected mass population of 8505C and of TPC-1 cells after stable transfection with TUSC2 or Control Vector (C.Vector) plasmid. (**b**) MTS assay was performed to evaluate the proliferation rate in 8505C (left) and TPC-1 (right) cell lines after stable TUSC2 transfection. Absorbance at O.D. 490 nm was measured every 24 h for four days. Data represent the mean of three experiments ± standard errors. (**c**) Colony formation ability of 8505C cells stably transfected with TUSC2 or with Control Vector (C. vector) was examined by soft agar assay to determine anchorage-independent growth. Cells were cultured in semisolid medium and after three weeks of plating, the colonies were imaged (left) and counted (right) under a microscope at 10x magnification. The experiment was performed in triplicate. ND: Not detected.

To determine the effects of TUSC2 on thyroid cancer aggressiveness, we stably transfected the TUSC2 plasmid or the corresponding control (indicated as Control Vector) into anaplastic (8505C) and papillary (TPC-1) thyroid cancer cell lines. A mass population for each cell line, expressing high levels of TUSC2, was selected and utilized (Figure 1a).

First, we investigated the effects of forced expression of TUSC2 on the cell proliferation rate and on cell cycle progression. As shown in Figure 1b, the methyl tetrazolium compound (MTS) assay demonstrated that TUSC2 reduced the proliferation of 8505C (left) and TPC-1 (right) cells compared to the corresponding controls. 

Additionally, the effect of TUSC2 on the aggressiveness of thyroid cancer cells was measured using a soft agar clonogenic assay. Figure 1c revealed that forced expression of TUSC2 dramatically decreased the growth rate of 8505C cells in soft agar.

**Figure 2:**
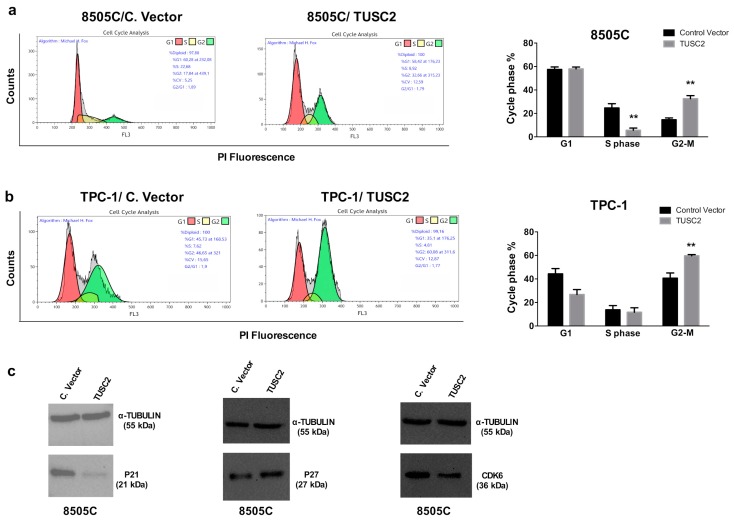
TUSC2 forced expression reduced thyroid cancer cell proliferation. Flow cytometry analysis of the cell cycle distribution in 8505C (**a**) and TPC-1 (**b**) cells stably transfected with TUSC2 or with Control Vector (C. Vector) plasmids. A representative fluorescence-activated cell sorting (FACS) plot is shown in the left panel while the average of two independent experiments is shown on the right. ** *p* < 0.01. Error bars indicate standard errors. (**c**) Western blot of p21, p27, CDK6 and TUBULIN in 8505C/C. vector and 8505C/TUSC2 cells.

We analysed cell cycle progression in 8505C and TPC-1 transfected cells by flow cytometry with propidium iodide (PI) staining. Accordingly, we found that TUSC2 expression caused accumulation of the cells in the G2/M phase (Figure 2a,b). Furthermore, Western blot analysis was performed to analyse the expression level of several cell cycle regulators, and the results showed that p21 WAF1/CIP1 and CDK6 were decreased, while p27/KIP1 expression was increased in 8505C/TUSC2 cells compared to the control (Figure 2c). Therefore, TUSC2 restoration could represent an important tool to arrest cancer cell proliferation.

### 2.2. TUSC2 Forced Expression Decreased the Migration and Invasion of Thyroid Cancer Cells

Cell migration and invasion ability are two essential steps in tumour metastasis, thus migration and invasion were analysed after stable TUSC2 transfection in thyroid cancer cell lines by wound healing and Matrigel matrix assays.

**Figure 3:**
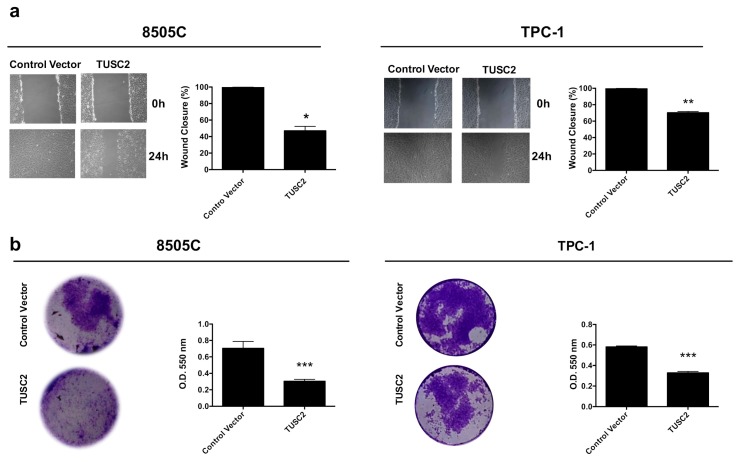
TUSC2 forced expression reduced thyroid cancer cell motility. (**a**) A wound was introduced on a confluent monolayer of 8505C (left) and TPC-1 (right) cells stably transfected with TUSC2 plasmid or Control Vector, and cell migration into the wound was monitored for 24 h. Images were taken at 10× magnification. Wound closure was measured by calculating pixel densities in the wound area and expressed as percentage ± standard errors. (**b**) Stably transfected 8505C (left) and TPC-1 (right) cells were plated on a Matrigel matrix and allowed to invade the Transwell insert for 24 h. Invaded cells were stained, photographed and quantified by measuring the absorbance at O.D. 550 nm. Bars indicate the mean of duplicate experiments ± standard errors. Asterisks indicate * *p* < 0.05, ** *p* < 0.01 and *** *p* < 0.001.

We found that 8505C/TUSC2 and TPC-1/TUSC2 cells showed less wound closure than cells transfected with the Control Vector at the same time point (Figure 3a).

Moreover, as shown in Figure 3b and in the relative quantification, the number of invaded cells on the surface of the Transwell coated with Matrigel matrix was lower in TPC-1 and in 8505C cells overexpressing TUSC2 than in cells transfected with the Control Vector.

The obtained results clearly indicate that TUSC2 restoration decreased the migration and invasion of thyroid cancer cell lines.

### 2.3. TUSC2 Forced Expression Increased Sensitivity to Apoptosis Induced by Doxorubicin and Staurosporine in Thyroid Cancer Cells

We have previously reported that TUSC2 rescues the resistance to apoptosis induced by its negative regulator, miR-584, in thyroid cancer cells [19]. Here, we explored the effects of TUSC2 alone and after treatments with two different apoptotic agents, staurosporine and doxorubicin, in TPC-1 and in 8505C cells.

**Figure 4:**
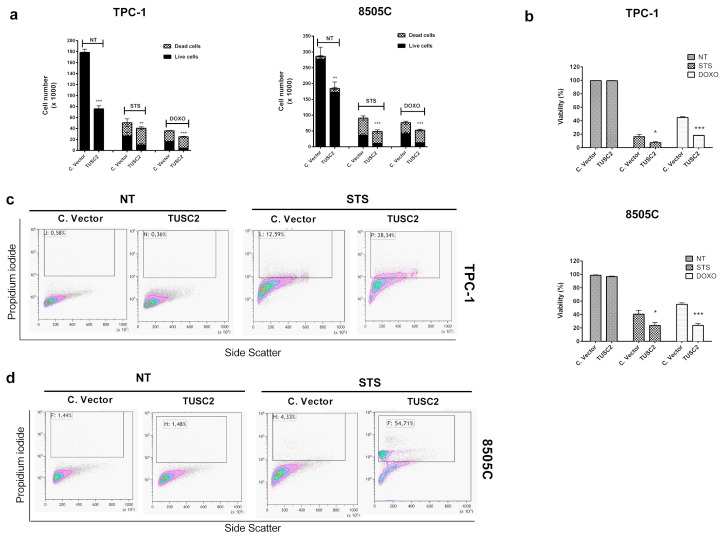
TUSC2 forced expression increased sensitivity to apoptosis induced by doxorubicin (DOXO) and staurosporine (STS) in thyroid cancer cells. 8505C and TPC-1 cells stably transfected with TUSC2 or Control Vector plasmids were treated with 1 µM of doxorubicin (DOXO) or 2.5 µM of staurosporine (STS). After 48 (for DOXO) and 24 h (for STS), cells were collected by trypsinization, stained for 10 min with trypan blue and counted in triplicate. Histograms show the number of live and dead cells (**a**) and the percentage of cell viability (**b**) ± standard errors. Stably-transfected TPC-1 (**c**) and 8505C (**d**) cells were treated with 2.5 µM of STS for 6 h and the percentage of apoptotic cells was measured by flow cytometry with propidium iodide (PI) staining. Asterisks indicate * *p* < 0.05, ** *p* < 0.01 and *** *p* < 0.001.

To this aim, transfected cells were treated with doxorubicin (1 µM) or with staurosporine (2.5 µM) and counted with trypan blue after 48 and 24 h, respectively. As shown in Figure 4a,b, treatments with staurosporine and doxorubicin in 8505C/TUSC2 and TPC-1/TUSC2 cells reduced the cell number (a) and cell viability (b) in comparison to that in control cells.

Finally, we analysed apoptosis in transfected cells treated with staurosporine by flow cytometry with propidium iodide staining. Figure 4c,d shows that the percentage of apoptotic cells in 8505C/TUSC2 and in TPC-1/TUSC2 cells, respectively, treated with staurosporine was increased compared to that in the corresponding controls. On the other hand, forced expression of TUSC2 in untreated (NT) TPC-1 and 8505C cells did not induce apoptosis (Figure 4c,d left).

### 2.4. TUSC2 Increased SMAC/DIABLO and CYTOCHROME C Protein Expression in Response to Apoptotic Stimuli in Thyroid Cancer Cells

In an attempt to identify the regulatory networks primarily involved in TUSC2 signalling activity, the Proteome Profiler Human Apoptosis Array (R&D Systems) was performed for simultaneous detection of 43 human apoptotic markers. To this aim, we assessed the expression levels of these apoptosis-related proteins by Western blot in 8505C/TUSC2 and control cells following staurosporine treatment (2.5 µM for 24 h).

**Figure 5:**
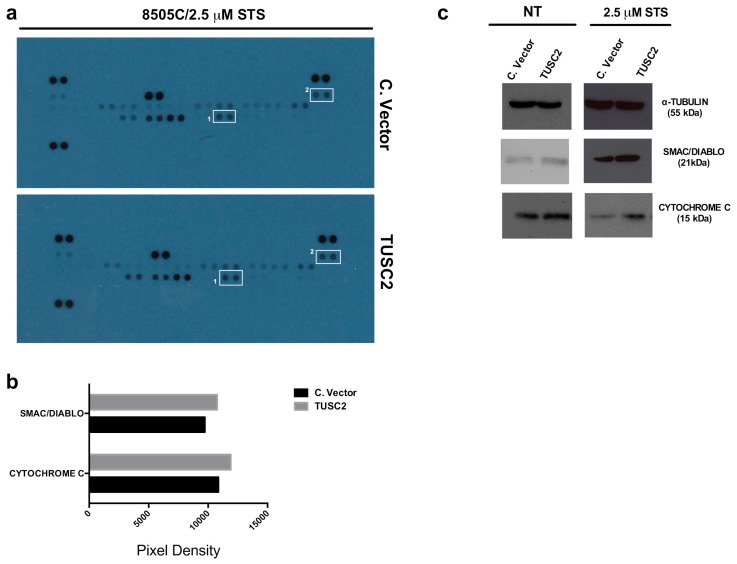
TUSC2 forced expression increased SMAC/DIABLO and CYTOCHROME C protein levels. (**a**) 8505C cells stably transfected with TUSC2 or with Control Vector (C. Vector) were treated for 24 h with 2.5 µM STS, and then 400 µg of protein lysates were blotted on the Proteome Profiler Human Apoptosis Array nitrocellulose membrane purchased from R&D Systems. Spots highlighted and labelled as #1 refer to SMAC/DIABLO and as #2 refer to CYTOCHROME C proteins. (**b**) Quantification of pixel intensities was performed with the ImageJ software programme (version 1.50i) and each protein is represented as the average signal (pixel density) of a pair of duplicate spots. Pixel density was normalized by subtracting the average background signal. (**c**) Western blot for SMAC/DIABLO, CYTOCHROME C and TUBULIN proteins in an independent cell lysate of transfected 8505C cells untreated (NT) or treated with STS.

Figure 5a shows that in 8505C/TUSC2 cells treated with staurosporine, the expression levels of the apoptotic proteins SMAC/DIABLO and CYTOCHROME C were changed compared to those in control cells. The quantification of pixel intensities is shown in Figure 5b.

Additionally, in an independent Western blot, we confirmed the upregulation of these proteins in 8505C/TUSC2 cells compared to 8505C/C. vector following the treatment with staurosporine (Figure 5c, right). This result was also observed to a lesser extent in untreated 8505C/TUSC2 (NT) cells (Figure 5c, left).

### 2.5. TUSC2 Silencing Increased the Malignant Phenotype of the Nthy-ori 3-1 Cell Line

**Figure 6:**
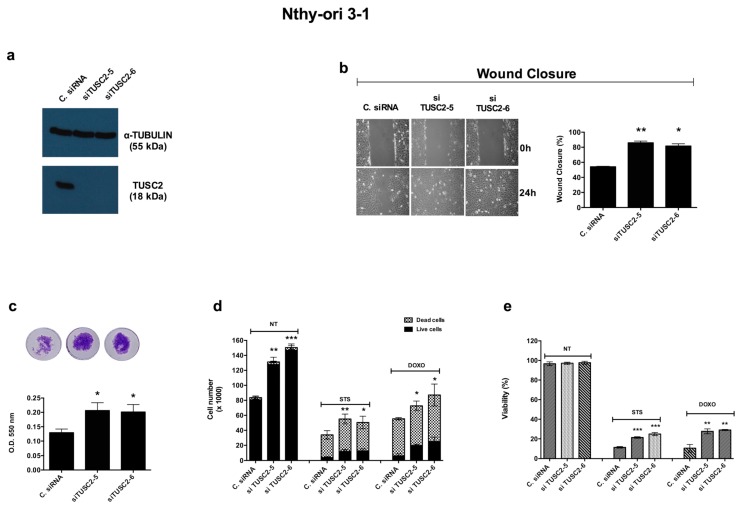
TUSC2 silencing increased the malignant phenotype of the Nthy-ori 3-1 cell line. (**a**) Nthy-ori 3-1 cells were transiently transfected with two different siRNAs (siTUSC2-5 or siTUSC2-6) or with Control siRNA (named C. siRNA). After 72 h, protein lysates were subjected to immunoblotting with the indicated antibodies. (**b**) Twenty-four hours post transfection, a wound was generated on a confluent monolayer of Nthy-ori 3-1 transfected cells. Cells were imaged (left) under a microscope at 10x magnification and the wound closure distance was quantified by calculating pixel densities in the wound area and expressed as the percentage of wound closure (right). (**c**) The day after the transient transfection, cells were seeded in the upper chambers of the Transwell and allowed to invade the Matrigel matrix for 24 h. Invasion ability is expressed as absorbance at O.D. 550 nm. (**d**,**e**) The Nthy-ori 3-1 cell line was transiently transfected with the indicated siRNA and, the day after, the cells were treated with 1 µM of DOXO (for 48 h) or with 2.5 µM of STS (for 24 h). At the end of the treatment, cells were stained with trypan blue and counted (**d**) and the percentages of cells viability (**e**) were determined. Values represent the average of duplicate experiments ± standard errors. * *p *< 0.05, ** *p* < 0.01 and *** *p *< 0.001.

To confirm the previous results, we performed the opposite experiment using two different TUSC2 siRNAs (no. 5 and 6) to downregulate the endogenous level of this tumour suppressor in the Nthy-ori 3-1 cell line. The silencing efficiency was evaluated by Western blot 72 h after transient transfection (Figure 6a).

We evaluated cell motility in silenced cells compared to control cells. Silencing of TUSC2 increased the migration (Figure 6b) and invasion (Figure 6c) of Nthy-ori 3-1 cells compared to cells transiently transfected with Control siRNA.

Finally, in order to verify the results obtained for the role of TUSC2 in the apoptotic process of thyroid cancer cells, a trypan blue assay was performed in Nthy-ori 3-1 cells transfected with siRNAs targeting TUSC2 (siRNA TUSC2-5, siRNA TUSC2-6) or with Control siRNA and treated with doxorubicin and staurosporine. Silencing of TUSC2 in treated cells induced resistance to apoptosis by increasing the cell number (Figure 6d) and cell viability (Figure 6e) compared to those of the corresponding control cells.

### 2.6. TUSC2 Effects Are Partially Mediated by SMAC/DIABLO

To further gain insight into the molecular mechanisms induced by TUSC2 in thyroid cancer cells, we focused on the role of SMAC/DIABLO.

We initially asked whether TUSC2 is able to directly bind SMAC/DIABLO by using a proximity ligation assay (PLA) assay, which allows in situ detection of protein interactions. As shown in the Figure S1, we found PLA signals, detected by flow cytometry, only in the positive control, while PLA signals were absent in 8505C/TUSC2 cells untreated or treated with staurosporine, suggesting that the induction of SMAC/DIABLO by the TUSC2 protein occurs indirectly through the binding with another protein.

**Figure 7:**
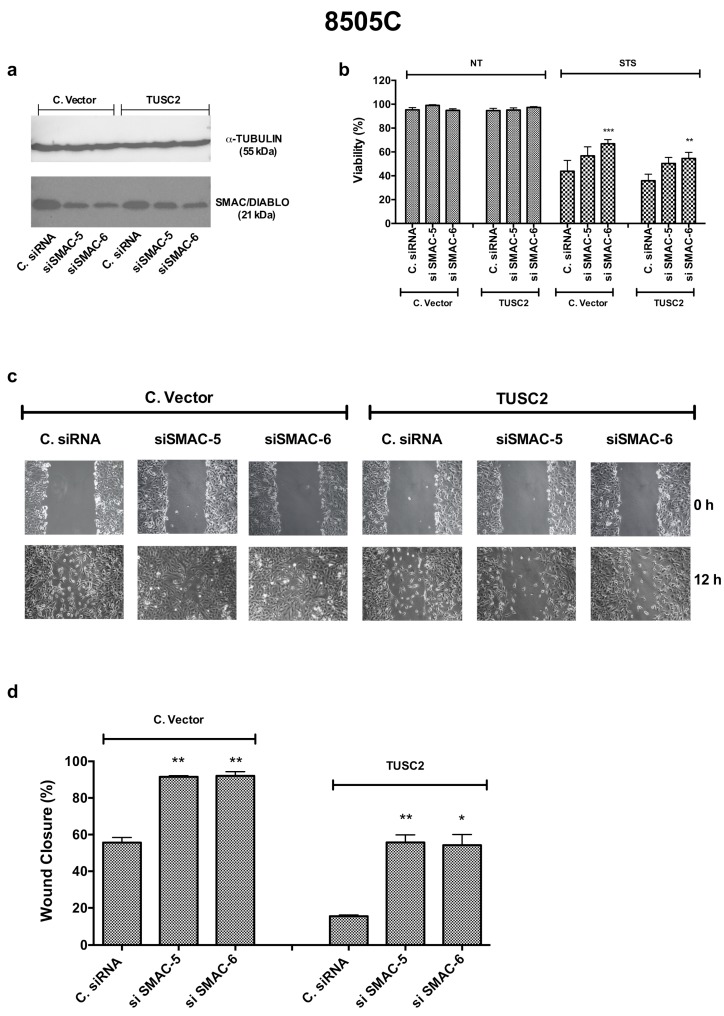
Silencing SMAC/DIABLO partially rescues the TUSC2-induced phenotype in the 8505C cell line. (**a**) 8505C/C. vector and 8505C/TUSC2 cells were transiently transfected with two different SMAC/DIABLO siRNAs (siSMAC-5 or siSMAC-6) or with Control siRNA (named C. siRNA). After 72 h, protein lysates were subjected to immunoblotting with the indicated antibodies. (**b**) 8505C/C. vector and 8505C/TUSC2 cell lines were transiently transfected with the indicated siRNA and the day after, the cells were treated with 2.5 µM of STS for 24 h. At the end of the treatment, cells were stained with trypan blue and counted. (**c**–**d**) 8505C/C. vector and 8505C/TUSC2 cell lines were transiently transfected with the indicated siRNA and, 24 h post transfection, a wound was generated on the confluent monolayer of 8505C/C. vector and 8505C/TUSC2 transfected cells. Cells were imaged under a microscope at 10 x magnification (**c**) and the wound closure distance was quantified by calculating pixel densities in the wound area and expressed as the percentage of wound closure (**d**). Values represent the average of duplicate experiments ± standard errors. * *p* < 0.05, ** *p* < 0.01 and *** *p* < 0.001.

Then, we asked whether silencing of SMAC/DIABLO in 8505C/TUSC2 and control cells could restore the biological phenotype of TUSC2. Figure 7a shows the silencing efficiency of SMAC/DIABLO (by using two different siRNAs, 5 and 6) in 8505C/TUSC2 and 8505C/C. vector cells. As shown in Figure 7b, silencing SMAC/DIABLO partially rescued the effects induced by TUSC2 on cell viability after staurosporine treatment. Additionally, Figure 7c,d shows that silencing SMAC/DIABLO partially rescued the effects induced by TUSC2 on cell migration.

**Figure 8:**
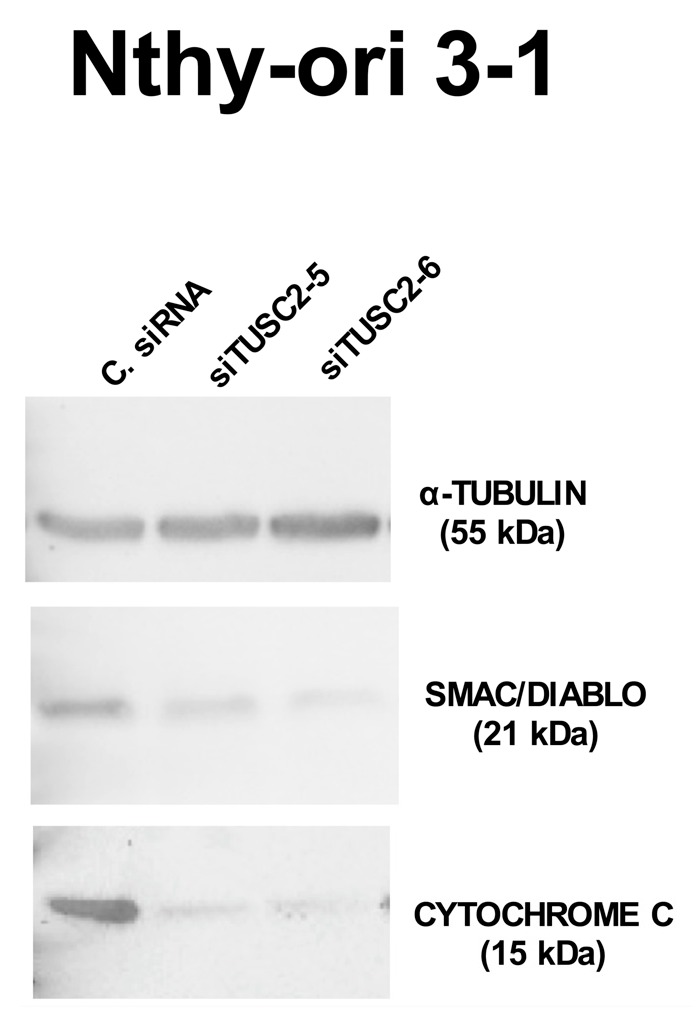
TUSC2 silencing decreased expression levels of SMAC/DIABLO and of CYTOCHROME C in the Nthy-ori 3-1 cell line. Nthy-ori 3-1 cells were transiently transfected with two different siRNAs (siTUSC2-5 or siTUSC2-6) or with Control siRNA (named C. siRNA). After 72 h, protein lysates were subjected to immunoblotting with the indicated antibodies.

Finally, we confirmed these results also in Nthy-ori 3-1 cells. We first evaluated the expression levels of the identified apoptotic proteins after TUSC2 silencing and found that, in Nthy-ori 3-1 cells silenced with both TUSC2 siRNAs, SMAC/DIABLO and CYTOCHROME C levels decreased compared with those in cells transfected with control siRNA (Figure 8).

**Figure 9:**
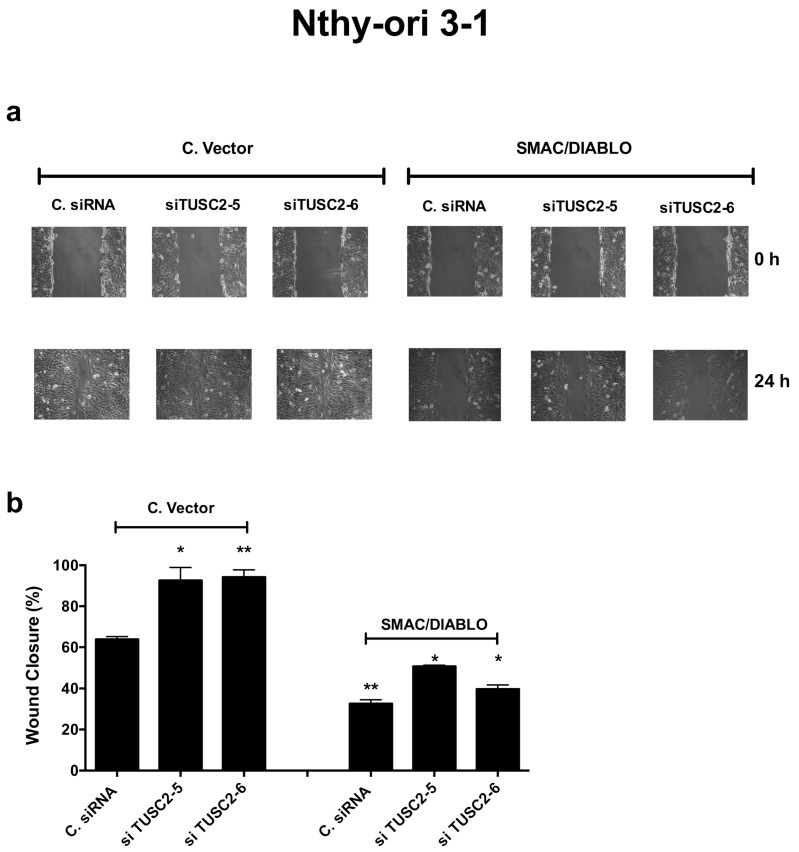
SMAC/DIABLO partially rescued the TUSC2 induced phenotype in the Nthy-ori 3-1 cell line. Nthy-ori 3-1 cells were transiently transfected with two different siRNAs (siTUSC2-5 or siTUSC2-6) or with Control siRNA (named C. siRNA) for 24 h, and then transfected with a SMAC/DIABLO plasmid or Control Vector. Then a wound was generated on the confluent monolayer of transfected cells. Cells were imaged under a microscope at 10x magnification (**a**) and the wound closure distance was quantified and expressed as the percentage of wound closure (**b**). Values represent the average of duplicate experiments ± standard errors. * *p* < 0.05 and ** *p* < 0.01.

Next, we performed a rescue experiment: Nthy-ori 3-1 cells were transiently silenced for TUSC2 and then transfected with SMAC/DIABLO or Control Vector and the migration ability was evaluated by a wound healing assay. As shown in Figure 9, forced expression of SMAC/DIABLO reversed the motility effects induced by silencing of TUSC2 in Nthy-ori 3-1 cells.

Overall, these results suggest that SMAC/DIABLO is a mediator of TUSC2 biological effects in thyroid cancer cells.

## 3. Discussion

Thyroid cancer is the most frequent cancer of the endocrine glands with an increasing incidence worldwide due mainly to improved resolution of imaging detection [17,20]. Papillary thyroid cancer (PTC), derived from follicular cells, represents the most frequent histological subtype among well-differentiated thyroid cancers. Although this carcinoma is characterized by an excellent outcome, patients with invasive or metastatic disease have poorer overall survival and become resistant to therapies [17].

Anaplastic thyroid cancer (ATC) is one of the most aggressive human cancers; it can be derived from normal thyroid follicular cells (thyrocytes) or directly from well-differentiated thyroid cancer. ATC cases are generally incurable as they present a high rate of distant metastasis leading to death in less than one year from diagnosis; fortunately, the percentage of ATC cases is very low [15,21,22].

Although recently there has been a great progress through next-generation sequencing, in understanding the molecular signatures of metastatic thyroid tumours [23], novel treatment approaches are urgently needed for these tumours.

We have previously showed a negative association between the expression of TUSC2 and thyroid tumour progression [19].

Since many studies have described a significant impact of the loss or reduction of the TUSC2 tumour suppressor on different cancer types [2], the aim of this study was to deepen the knowledge on the molecular mechanisms modulated by this tumour suppressor gene in thyroid cancer cells.

Here, we report that TUSC2 restoration inhibits tumour cell growth by arresting the cell cycle progression and by reducing the motility phenotype of thyroid cancer cell lines. Importantly, we have obtained preliminary evidence that, in mouse xenografts, tumours formed by ATC overexpressing TUSC2 cells are smaller compared to the control (unpublished results).

Our data are consistent with the study by Kondo and colleagues that reported that FUS1 (TUSC2) overexpression decreases colony formation and was associated with an alteration of cell cycle kinetics in a FUS1-inducible system of lung cancer cells. More recently, Li and colleagues reported that the coexpression of the tumour suppressor genes, TUSC2 and LKB1, leads to an inhibition of lung cancer cell growth inducing an arrest of the cell cycle [24,25].

TUSC2 has pro-apoptotic activity in human lung cancer cells through the regulation of the protein kinases EGFR, PDGFR, AKT, c-ABL, and c-KIT [3,10], through the activation of the STAT1 signal pathway and through regulation of the P53 protein [26].

Here, in an attempt to identify the regulatory networks of TUSC2-induced apoptosis in thyroid cancer cells, we used the Proteome Profiler Human Apoptosis Array (R&D Systems), and we found an increase in SMAC/DIABLO and CYTOCHROME C proteins in cells transfected with TUSC2 in response to apoptotic stimuli.

Consistent with our data, in the literature it has been reported that, in a TUSC2 knockout mouse model, cells have lower levels of CYTOCHROME C [27].

Thus, since the role of TUSC2 on the CYTOCHROME C protein has already been studied [27], we focused on SMAC/DIABLO and investigated the effects of this protein on the TUSC2 biological phenotype.

Here, we obtained evidence that TUSC2 induction of the SMAC/DIABLO protein is indirect and likely occurs through binding to another protein that subsequently directly binds to SMAC/DIABLO. Importantly, we found that the modulation of SMAC/DIABLO rescued the phenotype induced by TUSC2 protein in thyroid cell lines.

In this regard, it is worth noting that both proteins are localized in the mitochondria. Indeed, SMAC/DIABLO is a mitochondrial protein that promotes CYTOCHROME C-dependent CASPASE activation and apoptosis [28]. Additionally, TUSC2 induces apoptosis through the activation of the intrinsic mitochondrial-dependent pathway, and these data were also confirmed by the fact that APAF-1 is one of the cellular targets of the TUSC2 protein by its direct protein–protein interactions [3,29].

Interestingly it has been demonstrated that, in thyroid cancer cells, resistance to chemotherapeutic agents requires low levels of SMAC/DIABLO [30].

Several studies have shown that overexpression of SMAC/DIABLO can sensitize cancer cells to apoptosis, thus the development of small-molecule SMAC mimetics has been an attractive goal for cancer treatment in the last decade [30,31,32].

The mechanisms responsible for TUSC2 downregulation are partially known. Notably, the TUSC2 gene is located on human chromosome 3p21.3, a region found to be homozygously deleted in early events of the development of breast and lung cancer, suggesting that the genes located in this deleted region could function as gatekeepers in the molecular pathogenesis of these tumours. However, it is possible that in other human cancers in which this deletion did not occur, other mechanisms cause a reduction in TUSC2 expression. In fact, in the literature it has been reported that several microRNAs (miRNAs) are implicated in this process. The oncogenic activities of miR-93, miR-98 and miR-197 in lung cancer are mediated by the silencing of TUSC2 [33]. In ovarian and nasopharyngeal cancers it has been reported that miRNA-663 promotes cell growth, migration and invasion by inhibiting TUSC2 [34,35].

Additionally, miR-378 promotes cell survival, tumour growth and angiogenesis by targeting TUSC2 and miR-378/TUSC2 levels predict adverse prognosis in acute myeloid leukaemia patients [36,37].

Likewise, in thyroid cancer, we have reported that the overexpression of miR-584 induces a reduction in TUSC2 by directly targeting its 3′untranslated region [19].

In conclusion we provide new evidence on the importance of the TUSC2 tumour suppressor in thyroid carcinogenesis. Notably, our findings indicate the possibility that restoration of TUSC2 expression, combined with specific pro-apoptotic stimuli, could represent a cancer strategy for patients affected by aggressive forms of thyroid carcinoma.

## 4. Materials and Methods

### 4.1. Cell Cultures

An anaplastic thyroid cancer cell line (8505C) and papillary thyroid cancer cell line (TPC-1) were grown in Dulbecco’s modified Eagle’s medium (DMEM) (Thermo Fisher Scientific, Waltham, MA, USA), while the normal immortalized human primary thyroid follicular epithelial cell line (Nthy-ori 3-1) was grown in Roswell Park Memorial Institute 1640 (RPMI) medium. All growth media used were supplemented with 10% foetal bovine serum (FBS), l-glutamine and penicillin/streptomycin (Thermo Fisher Scientific).

### 4.2. Cell Transfections

For stable cell generation, TPC-1 and 8505C cells were transfected with 4 µg of TUSC2 plasmid or with the corresponding Control Vector purchased from GeneCopoeia (Nivelles, Belgium) using Lipofectamine 2000 (Thermo Fisher Scientific) according to the manufacturer’s instructions. After 48 h, transfected cells were selected with geneticin (Sigma-Aldrich, St. Louis, MO, USA) to obtain several cell clones and mass populations. In these cells the level of TUSC2 was assayed by Western blot and one mass population for each cell line with the highest TUSC2 level was used for functional experiments.

8505C/TUSC2 and C. Vector were also transiently transfected with specifics siRNAs for SMAC/DIABLO, named siSMAC-5 (catalogue number SI02655576) and siSMAC-6 (catalogue number SI00299999) (Qiagen, Hilden, Germany), using HiPerFect (Qiagen) for 24 h according to the manufacturer’s instructions.

The Nthy-ori 3-1 cell line was transiently transfected with 5 µl of 100 µM of small interfering RNA (FlexiTube siRNA Qiagen) specific for TUSC2, named TUSC2-5 (catalogue number SI02664606) and TUSC2-6 (catalogue number SI0266461), or with negative control siRNA (AllStars Negative Control siRNA; SI03650318) using HiPerFect Transfection reagent (Qiagen) according to the manufacturer’s instructions.

Where indicated after transient transfection, the Nthy-ori 3-1 cell line was transfected with SMAC/DIABLO (EX-U1000-M43) plasmid purchased from GeneCopeia (Tebu-bio) using Lipofectamine 2000 (Thermo Fisher Scientific) according to the manufacturer’s instructions.

### 4.3. Western Blot

Extracted proteins were quantified using a spectrophotometer (Bio-Rad, Hercules, CA, USA) and a Bradford assay (Bio-Rad), and the lysates were subjected to sodium dodecyl sulphate–polyacrylamide gel electrophoresis (SDS-PAGE) according to standard procedures. The nitrocellulose membranes were hybridized with the rabbit polyclonal anti-TUSC2 (ab246970, Abcam, Cambridge, UK) diluted 1:200; mouse monoclonal anti-SMAC/DIABLO (clone 3A9.1) (Millipore, Danvers, MA, USA) diluted 1:1000; mouse monoclonal anti-CYTOCHROME C (sc-13156) (Santa Cruz, Dallas, TX, USA) diluted 1:500; rabbit monoclonal anti-p21 WAF1/CIP1 (12D1) diluted 1:1000 (Cell Signalling); rabbit monoclonal anti-p27 KIP1 (D69C12) diluted 1:1000 (Cell Signalling); mouse monoclonal anti-CDK6 (DCS83) diluted 1:1000 (Cell Signalling) and with mouse monoclonal anti-α-TUBULIN (Sigma-Aldrich) diluted 1:10000 antibodies. Secondary anti-mouse and anti-rabbit antibodies coupled to horseradish peroxidase were purchased from Bio-Rad and diluted 1:3000. Immune complexes were visualized by an enhanced chemiluminescence detection kit (ECL, Thermo Fisher Scientific). Image Lab^TM^ Software (Bio-Rad) was used to detect and analyse bands intensities automatically.

### 4.4. Migration and Invasion Assays

Migration and invasion abilities were assessed by wound healing and by Matrigel matrix assays, respectively, as previously described [38].

In brief, for the wound healing experiment, cells were plated in 6-well culture plates and grown to confluence. A scratch was generated on a confluent cell monolayer and the closing of the wound was monitored and measured using Cell^a^ software (Olympus Biosystem Gmb, London, UK).

For the invasion assays, transfected cells were seeded into the upper Transwell chambers precoated with Matrigel matrix (BD Biosciences, San Jose, CA). After incubation for 24 h, cells that had invaded through the chamber membrane were fixed with 11% glutaraldehyde solution (Sigma-Aldrich) for 90 min, stained with crystal violet solution and, after elution, quantified by spectrophotometer measuring optical density (O.D.) 550 nm.

Before the wound healing and Matrigel matrix assays, cells were treated with mitomycin (Sigma-Aldrich) to inhibit cell proliferation during these experiments.

### 4.5. MTS Assay

Cell proliferation was measured by the 3-(4,5-dimethylthiazol-2-yl)-5-(3-carboxy-methoxyphenyl)-2-(4-sulfophenyl)-2H-tetrazolium (MTS) colorimetric assay (CellTiter 96, Promega, Madison, WI, USA) according to the manufacturer’s instructions. TPC-1 and 8505C cells (1 × 10^3^), stably transfected with TUSC2 plasmid or with Control Vector, were plated in triplicate into 96-well culture plates. After 24, 48, 72 and 96 h, the cells were incubated with 20 μL of MTS reagent for 30 min. The quantity of formazan produced was determined by measuring optical density (O.D.) 490 nm using a microplate reader (Model 550, Ultramark Microplate Reader, Bio-Rad).

### 4.6. Trypan Blue Assay

For cell viability measurements, a trypan blue assay was performed. To this aim, transfected cells were plated in a 6-well plate. The day after plating, cells were treated with 1 µM doxorubicin (Sigma-Aldrich) or with 2.5 µM staurosporine (Sigma-Aldrich) for 48 or 24 h, respectively. After treatment, cells were collected by trypsinization and stained with 0.4% trypan blue reagent (Bio-Rad) according to the manufacturer’s instructions. Each count was performed in triplicate using TC20^TM^ Automated Cell Counter (Bio-Rad). By trypan blue staining, cell viability (expressed as percentage %) was calculated by dividing the number of unstained (viable) cells by the number of total cells (stained and unstained).

### 4.7. Anchorage-Independent Cell Growth in Soft Agar

Colony formation was determined by soft agar assay. Briefly, transfected 8505C cells (8 × 10^4^) were resuspended in DMEM containing 2% agar noble and plated onto the solidified noble agar deposited in 60 mm petri dishes. Cells were periodically observed under a microscope to monitor colony formation. Fresh medium was added every four days. After three weeks, colonies were counted under an optical microscope at 10× magnification and imaged. The experiment was performed in triplicate.

### 4.8. Flow Cytometry

Cell cycle progression and cell death were determined by fluorescence-activated cell sorting (FACS) analysis using propidium iodide staining and an FC500 Cytometer (Beckman Coulter, Milan, Italy) as previously described [38].

Briefly, for the cell cycle, stably transfected cells were stained with the DNA PREP Reagents Kit (Beckman Coulter) according to the manufacturer’s instructions, and a minimum of 10,000 single cell events were recorded using the CXP Software (Beckman Coulter). Then, cell cycle analysis was performed using Kaluza Analysis Software 2.1 (Beckman Coulter) with the Michael Fox algorithm to determine G1, S and G2/M phases [39].

For cell death studies, cells were treated with staurosporine (2.5 µM) for 6 h and incubated with 1 μL of a 10 μg/mL propidium iodide (PI) solution (Beckman Coulter) for FACS analysis.

### 4.9. Human Apoptosis Array

8505C cells stably transfected with the TUSC2 or control plasmids were treated with 2.5 µM of staurosporine (Sigma-Aldrich) for 24 h, after which they were resuspended in 100 µl lysis buffer (R&D Systems, Minneapolis, MN) and quantified using the Bradford method. Cell lysates (400 µg) were added to the array membranes “Proteome Profiler Human Apoptosis Array”, obtained from R&D Systems, that had been incubated for 1 h with a blocking buffer, following the manufacturer’s instructions. The lysates were incubated with the blocked membranes overnight at 4 °C, and then each array was subjected to several washes for 10 min. Then, array membranes were incubated with detection antibody cocktail and streptavidin-HRP buffer followed by three washes. Plots were detected with Chemi Reagent Mix (R&D Systems) and images were obtained after autoradiographic exposure. Each apoptosis-related protein and control antibodies were spotted on the membrane in duplicate (R&D Systems). Quantification of the array was performed with the ImageJ software programme (version 1.50i) and each protein is represented as the average signal (pixel density) of the pair of duplicate spots. Pixel density was normalized by subtracting the average background signals.

### 4.10. Proximity Ligation Assay

To further evaluate whether TUSC2 is able to modulate the expression of SMAC/DIABLO through direct binding, we performed a proximity ligation assay (PLA) (Merck KGaA, Darmstadt, Germany) that allows in situ detection of protein interactions [40].

For these experiments, 8505C cells, transfected with the Control Vector or TUSC2-overexpressing vector, were treated with 2.5 μM of staurosporine (STS) for 24 h. Subsequently the cells were fixed and permeabilized using a PerFix Expose Kit (B26976, Beckman Coulter). Anti-TUSC2 (ab246970, Abcam) and anti-SMAC/DIABLO (clone 3A9.1, Millipore) antibodies were added at a concentration of 1:50 in Buffer 3. After 30 min of incubation, two wash steps in 1× PBS were performed and the PLA protocol was applied according to the manufacturer’s instructions (DUO94002, DUO92001, DUO92005, Sigma Aldrich, Germany). PLA assays were acquired on a Cytoflex cytofluorimeter and fluorescence was recorded in the fluorescein isothiocyanate (FITC) channel. A positive control was obtained using the HL60 cell line and two antibodies against well-known interacting proteins NF-Kb (rabbit, 622602 BioLegend, USA) and IKB-a (mouse, 662402, BioLegend, USA) [41].

### 4.11. Statistical Analysis

All data are reported as the mean ± standard error (SE). All statistical analyses were performed using GraphPad Software version 6.0 (La Jolla, CA, USA). The differences between groups were assessed by two-tailed t test and the data were considered statistically significant when *p* < 0.05.

## Supplementary Materials

Supplementary materials can be found at https://www.mdpi.com/1422-0067/21/3/702/s1.
