# Supplementary material for: The TUSC2 Tumour Suppressor Inhibits the Malignant Phenotype of Human Thyroid Cancer Cells via SMAC/DIABLO Protein
Source: Int J Mol Sci. 2020 Jan 21;21(3):702. doi: 10.3390/ijms21030702 (PMC7037188; doi:10.3390/ijms21030702)
Supplement: Supplementary file 1 [file ijms-21-00702-s001.pdf]

Supplementary Information

Mariniello et al: "*The TUSC2 tumor suppressor inhibits the malignant phenotype of human thyroid cancer cells via SMAC/DIABLO protein*".

Supplementary Figure 1

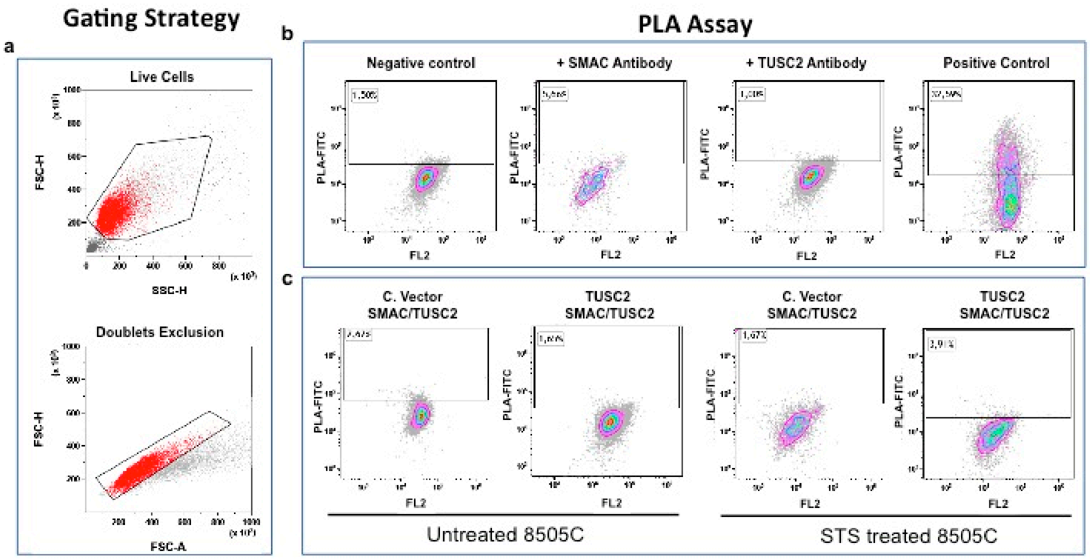

Supplementary Figure 1: SMAC DIABLO/TUSC2 interaction via Proximity Ligation Assay. Proximity ligation assay (PLA) experiments were conducted on active growing 8505C cells transfected with Control Vector or TUSC2 over-expressing vector. PLA signal associated with FITC fluorescence was recorded using a Cytoflex cytometer. In panel **a,** are reported the gating strategies. Panel **b** shows the PLA technical controls: Negative control was obtained using two PLA probes, anti TUSC2 and anti SMAC/DIABLO antibody alone. Positive control was obtained using HL60 cell line and two antibodies against well-known interacting proteins, NF-Kb and IKB-a. Panel **c** shows interaction analyses of TUSC2 and SMAC/DIABLO in untreated and staurosporine (STS) treated 8505C cells transfected with control vector or TUSC2-overexpressing vector. FITC-PLA vs FL2 density dot plots were reported for a better understanding of the results. Numbers show the percentage of positive cells respect to the negative control. Experiments were repeated twice with similar results.
